# Surmised total leucocyte counts miscalculate the parasite index of *Plasmodium vivax* malaria patients of tertiary and primary care settings in South-Western India

**DOI:** 10.1186/s12936-015-0669-4

**Published:** 2015-04-16

**Authors:** Kumar Rishikesh, Sathish Kitta Madivala, Prashantha Prabhu, Asha Kamath, Herikudru Ashok, Sudha Vidyasagar, Ananthakrishna Barkur Shastry, Kavitha Saravu

**Affiliations:** Department of Medicine, Kasturba Medical College, Manipal University, Manipal, Karnataka India; Department of Community Medicine, Kasturba Medical College, Manipal University, Manipal, Karnataka India; District Malaria Office, Udupi, Karnataka India

**Keywords:** Malaria, *Plasmodium vivax*, Parasite index, Parasite density, Assumed total leucocyte count, Total leucocyte count

## Abstract

**Background:**

For the calculation of parasite index (PI) by microscopy method, an assumed total leucocyte count (TLC) of 8,000/μL is used conventionally. However, due to obvious variation in the population and individual TLCs, use of 8,000/μL may result in either over/underestimation of the PI.

**Methods:**

This study was aimed at ascertaining the utility of 8,000/μL TLC, as well as other assumed TLCs, with respect to measured TLC for the calculation of PI. A tertiary care hospital and five primary health centres were the base for the prospective study conducted among microscopically proven, symptomatic *Plasmodium vivax* mono-infection patients aged ≥18 years. PIs calculated by assumed TLCs ranging from 4,000-11,000/μL were compared with those calculated by measured TLCs. Geometric mean with 95% confidence interval, Bland-Altman plot and Wilcoxon signed rank test were used for statistical analysis.

**Results:**

A total of 284 *P. vivax* mono-infection patients, including 156 from a tertiary care hospital and 128 from five primary health centres, were recruited in the study. Assumed TLCs below 5,000 cell/μL and above 5,500 cell/μL in tertiary care setting resulted in significant (p <0.05) underestimation and overestimation, respectively. However, in primary health centres, it was an assumed TLC of 5,000 cell/μL, below and above which there was significant (p <0.05) underestimation and overestimation observed, respectively.

**Conclusions:**

Assumed TLC of 8,000/μL is not suitable for the calculation of PI. Either actual TLC of the patient should be measured or a representative TLC should be derived for the population under investigation for any study requiring calculated PI by microscopy.

## Background

Parasite index (PI) is an essential malariometric marker for the precise monitoring of the efficacies of existing anti-malarial treatments, assessment of experimental anti-malarial drugs and evaluation of new diagnostic tests. An increased PI indicates disease severity and thereby guides the management in *Plasmodium falciparum* malaria [1]. Microscopy, being the ‘gold standard’ method for malaria diagnosis remains the mainstay of PI determination. The likelihood of microscopic detection of malaria parasite in a peripheral blood smear is a function of quantity of blood samples examined, duration of examination and level of expertise of individual microscopists. Initial reports [2,3] instituting the current method of PI calculation advocated the use of parasite/leucocyte ratio in the thick smear. A thick smear excludes the issue of heterogeneity in thickness of smears as it occurs with thin smears and holds about 20 times more volume of blood thus rendering it more sensitive than thin smears.

Considering the impracticability of estimation of individuals’ total leucocyte count (TLC) in surveys and in resource-poor settings, between 1950s and 1980s, an assumed value of 8,000 leucocytes/μL was suggested by the World Health Organization and others to be used for the determination of PI [2-4]. However, 8,000 leucocytes/μL is equivocal as it was merely based on studies held in Nigeria, West Africa. Indeed, depending upon the ethnicities, geographical locations and underlying morbidities affecting the level of leucocytaemia, PI may turn out to be either under or overestimation with respect to assumed 8,000 leucocytes/μL.

Of late, there have been a few studies from across the globe [5-7] denying the applicability of assumed TLC of 8,000/μL for PI calculation among respective populations. Surprisingly, there has been no study from India on this issue and the national guideline [8] advocates use of an assumed TLC of 8,000/μL for PI calculation. The current study was aimed at ascertaining the applicability of the assumed TLC of 8,000/μL for the calculation of PI among *Plasmodium vivax* mono-infection patients attending a tertiary care hospital and five primary health centres.

## Methods

### Study design and patients

The current manuscript is based on interim data of an in-progress prospective cohort study to assess the efficacy of anti-malarial drugs among microscopically confirmed, symptomatic *P. vivax* mono-infection patients aged ≥18 years attending a tertiary care hospital and five primary health centres. Patients who did not consent for study participation and had concomitant febrile illnesses were excluded. Further, *P. vivax* mono-infections were ascertained through nested polymerase chain reaction method [9].

### Ethics statement

Before the study commencement, approval from the institutional ethics committee of Kasturba Medical College and Kasturba Hospital, Manipal University, Manipal (IEC 193/2011) was obtained. A written informed consent was obtained from each participant prior to enrolment into the study and the identity of each participant was anonymized.

### Variables

Independent variables Participants’ TLC was measured on study recruitment in a haematology analyzer (Beckman Coulter LH 780). Besides measured TLCs, assumed TLCs ranging from 4,000–11,000/μL by an increment of 500/μL was used to calculate the on-recruitment PIs.

Dependent variable PI on recruitment was expressed as absolute number of asexual and/or sexual parasites present in 1 μL of peripheral blood. It was calculated as:

$$ PI\left(/\mu L\right)=\frac{PC}{WBC}\times TLC $$

Where, PI is parasite index, PC is the number of parasites counted in the blood smear, WBC is the number of white blood cells counted in the blood smear, TLC is the patients’ total leucocyte count (either ‘assumed’ or ‘measured’). Leishman’s stained peripheral blood smears were examined under Olympus CH20i microscope and PIs were determined independently by three microscopists. Using a manual tally counter, number of parasites and WBCs in corresponding microscopic fields were counted. If ten or more parasites were noted in up to 200 WBCs counts, further counting was stopped, otherwise counting continued up until 500 WBCs [8]. A mean of three consecutive PIs was used for further analysis.

### Statistical analysis

Patients’ age, fever duration, on admission TLC, and PIs were summarized as range, median (interquartile range), mean ± standard deviation, and 95% confidence interval (CI) of mean. Differences in mean age, fever duration, on recruitment TLCs, and PIs of tertiary care and primary care settings were compared by Mann–Whitney *U* test. Geometric mean, with its 95% CI for PIs, was determined to assess the relative differences by both measured and assumed TLCs. A 95% CI of geometric mean PI by assumed TLC, which did not overlap with that of measured one, was considered as significant difference. Bland-Altman plots were constructed to illustrate the agreement between the differences in the logarithmic means of the PIs. Proportion of patients having over/underestimated PI by assumed TLC was compared by Wilcoxon signed-rank test. Differences falling between −0.99 to 0.99 was defined as ‘exact estimation’, whereas, values below −1.00 and above 1.00 was considered to be overestimation and underestimation correspondingly. A p-value <0.05 was considered as statistically significant difference. Data analysis was done using Statistical Package for the Social Sciences version 15.0 (SPSS, South Asia, Bangalore, India). Geometric mean with 95% CI was determined using GraphPad Prism 5 for Windows, version 5.01 (^©^1992-2007 GraphPad Software, Inc).

## Results

A total of 284 *P. vivax* mono-infection patients, including 156 from a tertiary care hospital and 128 from five primary health centres, were recruited in the study. Male to female ratios were 140/16 and 118/10 in tertiary care hospital and primary health centres correspondingly.

### Baseline statistics

There was no statistically significant difference (p > 0.05) in the mean age, on recruitment TLCs and PIs of both the settings. Fever duration before presentation was significantly (p <0.05) longer for tertiary care centre than primary care. There was marked leucopaenia and leucocytosis among patients who attended tertiary care hospital, whereas, only leucopaenia was noted among primary health centres’ patients with upper range of TLC being within normal reference limit. Range of PI in tertiary hospital was markedly wider on either side than that of primary centres (Table 1).

**Table 1:** **Descriptive statistics of patients’ age, fever duration, on admission total leucocyte counts and parasite index**

	**Tertiary care hospital (N = 156)**				**Primary health centres (N = 128)**			
**Parameters**	**Age (years)**	**Fever duration (day)**	**Total leucocyte count (cells/μL)**	**Parasite index (/μL)**	**Age (years)**	**Fever duration (day)**	**Total leucocyte count (cells/μL)**	**Parasite index (/μL)**
**Range**	(18–76)	(1–60)	(1,600-35,000)	(22–31,040)	(18–75)	(1–10)	(1,400-9,000)	(62–17,712)
**Median (IQR)**	34 (24–46)	4 (3–6)	5,200 (4,200-6,400)	1,556 (524–3,617)	32 (25–45)	3(2–4)	4,900 (3,800-6,200)	1,520 (504–3,544)
**Mean ± SD**	36 **±** 14	5 **±** 4	5,686 **±** 3,301	1,367*	36 **±** 13	3 **±** 2	5,105 **±** 1,770	1,235*
**95% CI of mean**	34-38	4-6	5,168-6,204	1,106 – 1,689*	34-38	2.6-3.4	4,798-5,412	982 – 1,553*

CI = Confidence interval, IQR = interquartile range, SD = standard deviation, *Geometric mean and 95% CI of geometric mean.

### Degree of precision in PI by different TLCs

**Figure 1:**
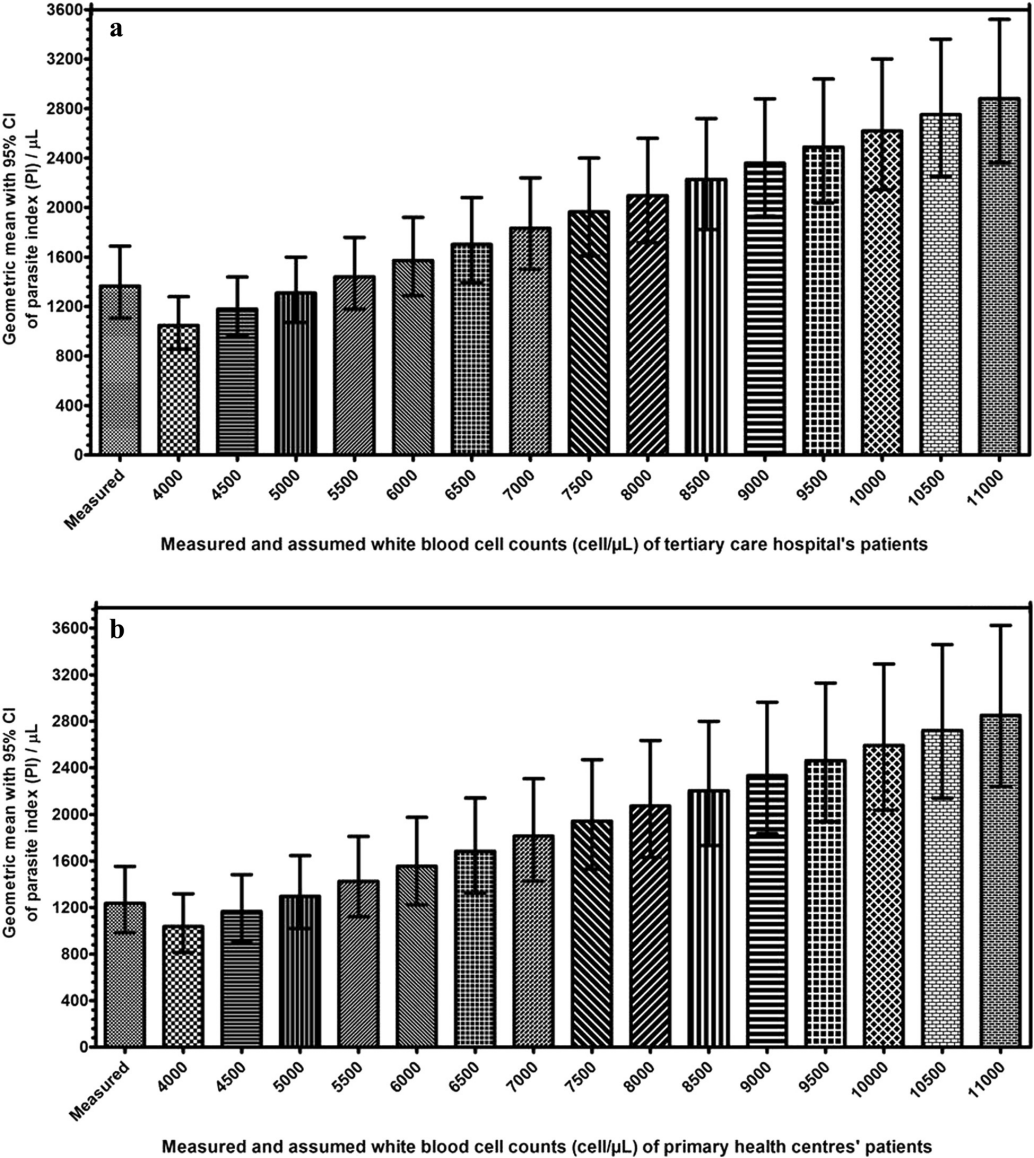
Column bar graph showing geometric mean with 95% confidence interval of geometric mean of the parasite index derived by measured and assumed total leucocyte count of patients attending **a)** tertiary care hospital and **b)** primary health centres. A 95% confidence interval of geometric mean PI by assumed TLC, which did not overlap with that of measured one, was considered as significant difference.

**Figure 2:**
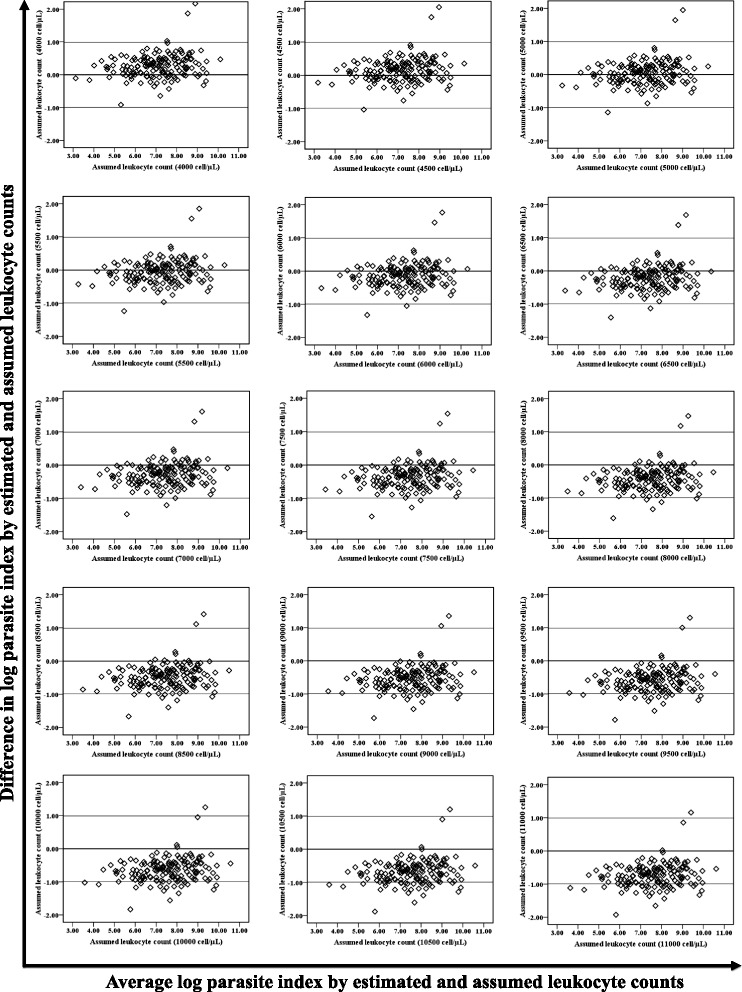
Bland-Altman plot showing parasite index of tertiary care setting by assumed and measured total leucocyte counts after logarithmic transformation.

**Figure 3:**
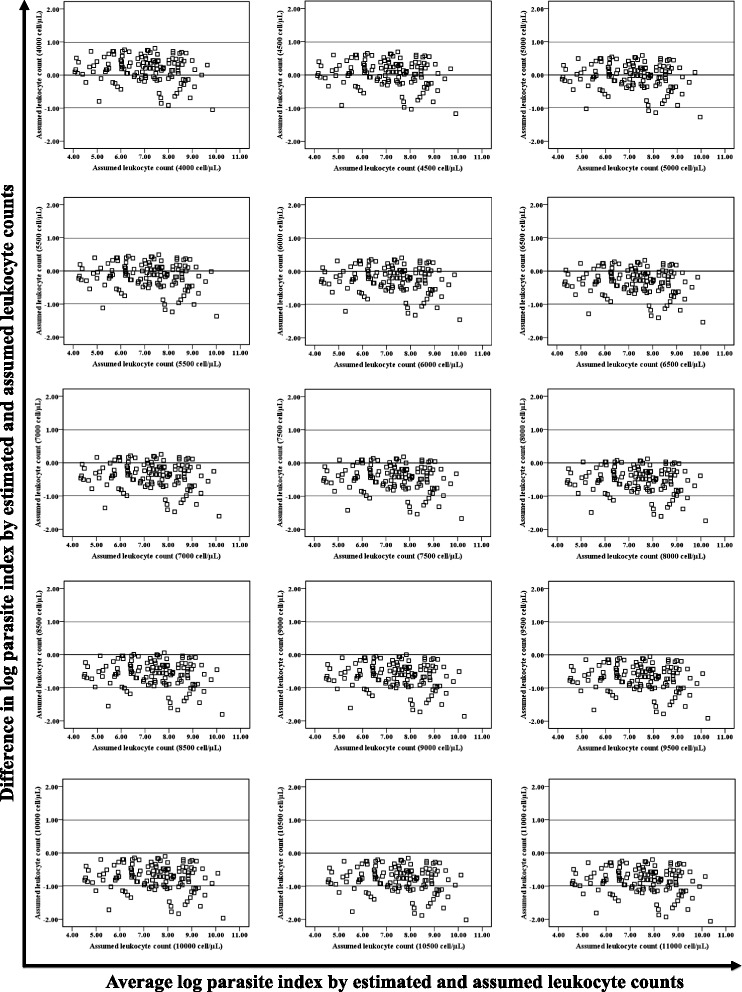
Bland-Altman plot showing parasite index of primary health centres by assumed and measured total leucocyte counts after logarithmic transformation.

There was no significant difference between the geometric mean PIs by assumed TLCs of 5,000 cell/μL and 5,500 cell/μL with that of measured TLCs in tertiary care hospital (Figure 1a), whereas in primary health centres, 95% CI of PIs by assumed TLCs of 4,500 cell/μL and 5,000 cell/μL appeared to have overlap with that of measured TLC, thus no significant difference (Figure 1b). Additionally, Bland-Altman plots showed agreement with results as appeared by geometric means for tertiary care (Figure 2) and primary care (Figure 3) settings. Further, Wilcoxon signed rank test reinforced statistically non-significant differences (p >0.05) in median PIs by assumed TLCs of 5,000 cell/μL and 5,500 cell/μL with that of measured one (Table 2) in tertiary care hospital. However, in primary health centres, PIs by assumed TLC of 5,000 cell/μL alone was found to have precise agreement (p >0.05) with that of measured one (Table 3). Assumed TLC below 5,000 cell/μL and above 5,500 cell/μL in tertiary care setting resulted in significant (p <0.05) under and overestimation, respectively. However, below and above only one intercept of 5,000 cell/μL, in primary health centres, significant (p < 0.05) under and overestimation was observed, respectively.

**Table 2:** **Comparison of degree of precision in parasite index by measured and assumed total leucocyte counts among** ***Plasmodium vivax*** **patients of a tertiary care hospital**

**Assumed TLC**	**Underestimation (%)**	**Precision (%)**	**Overestimation (%)**	**Parasite index by measured TLC [median (IQR), 1,556 (524, 3,617)]**	Z -score*	p-value*
4,000 cell/μL	1.9	98.1	0	1,150 (405, 2,585)	−7.5	<0.001
4,500 cell/μL	1.3	98.1	0.6	1,294 (456, 2,908)	−4.6	<0.001
**5,000 cell/μL**	**1.3**	**98.1**	**0.6**	**1,438 (506, 3,231)**	**−1.6**	**0.1** ^**¶**^
**5,500 cell/μL**	**1.3**	**98.1**	**0.6**	**1,581 (557, 3,554)**	**−1.1**	**0.29** ^**¶**^
6,000 cell/μL	1.3	97.4	1.3	1,725 (608, 3,878)	−3.6	<0.001
6,500 cell/μL	1.3	97.4	1.3	1,869 (658, 4,201)	−6.2	<0.001
7,000 cell/μL	1.3	97.4	1.3	2,013 (709, 4,524)	−7.6	<0.001
7,500 cell/μL	1.3	96.8	1.9	2,156 (759, 4,847)	−8.7	<0.001
8,000 cell/μL	1.3	95.5	3.2	2,300 (810, 5,170)	−9.5	<0.001
8,500 cell/μL	1.3	93.6	5.1	2,444 (861, 5,493)	−9.9	<0.001
9,000 cell/μL	1.3	91	7.7	2,588 (911, 5,816)	−10.1	<0.001
9,500 cell/μL	1.3	89.7	9	2,731 (962, 6,139)	−10.2	<0.001
10,000 cell/μL	0.7	88.2	11.2	2,875 (1,013, 6,463)	−10.2	<0.001
10,500 cell/μL	0.7	80.9	18.4	3,019 (1,063, 6,786)	−10.2	<0.001
11,000 cell/μL	0.6	76.3	23.1	3,163 (1,114, 7,109)	−10.3	<0.001

*Wilcoxon signed rank test, p-value <0.05 indicates statistically significant difference.

^¶^p-value >0.05 indicates no statistically significant difference, thus the respective assumed TLCs to have most robust precision with measured TLC for the calculation of parasite index.

Values in the assumed TLC row having robust precision with measured TLC are shown in bold face.

**Table 3:** **Comparison of degree of precision in parasite index by measured and assumed total leucocyte counts among** ***Plasmodium vivax*** **patients of five primary health centres**

**Assumed TLC**	**Underestimation (%)**	**Precision (%)**	**Overestimation (%)**	**Parasite index by measured TLC [median (IQR), 1,520 (504, 3,544)]**	Z - score*	p-value*
4,000 cell/μL	0	99.2	0.8	1,180 (365, 3,185)	−4.2	<0.001
4,500 cell/μL	0	98.4	1.6	1,328 (411, 3,583)	−2.0	0.04
**5,000 cell/μL**	**0**	**96.9**	**3.1**	**1,475 (456, 3,981)**	**−0.6**	**0.5** ^¶^
5,500 cell/μL	0	95.3	4.7	1,623 (502, 4,380)	−3.1	<0.002
6,000 cell/μL	0	94.5	5.5	1,770 (548, 4,778)	−5.1	<0.001
6,500 cell/μL	0	93	7	1,918 (593, 5,176)	−6.9	<0.001
7,000 cell/μL	0	92.9	7.1	2,065 (639, 5,574)	−7.9	<0.001
7,500 cell/μL	0	91.4	8.6	2,213 (684, 5,972)	−8.6	<0.001
8,000 cell/μL	0	89.8	10.2	2,360 (730, 6,370)	−9.4	<0.001
8,500 cell/μL	0	87.5	12.5	2,508 (776, 6,768)	−9.7	<0.001
9,000 cell/μL	0	85.2	14.8	2,656 (821, 7,166)	−9.8	<0.001
9,500 cell/μL	0	82.4	17.6	2,803 (867, 7,564)	−9.8	<0.001
10,000 cell/μL	0	77.2	22.8	2,950 (913, 7,963)	−9.8	<0.001
10,500 cell/μL	0	74	26	3,098 (958, 8,361)	−9.8	<0.001
11,000 cell/μL	0	71.1	28.9	3,245 (1,004, 8,759)	−9.8	<0.001

*Wilcoxon signed rank test, p-value <0.05 indicates statistically significant difference.

^¶^p-value >0.05 indicates no statistically significant difference, thus the respective assumed TLCs to have most robust precision with measured TLC for the calculation of parasite index.

Values in the assumed TLC row having robust precision with measured TLC are shown in bold face.

## Discussion

The present study examines the corroboration of a TLC of 8,000/μL and other assumed TLCs with measured TLC for the calculation of PI of *P. vivax* mono-infection patients attending a tertiary care setting and five primary health centres. At tertiary care centre assumed TLCs of 5,000/μL and 5,500/μL, whereas at primary health centres assumed TLC of 5,000/μL were found to have better corroboration for the calculation of PI than assumed TLC of 8,000/μL. Assumed TLCs of 5,000/μL and 5,500/μL are very close to the actual median WBC count of study population (Table 1). Despite significantly longer fever duration and marked leucocytosis in patients of tertiary care centre, there was no difference found between the PIs of the two settings. Corroborating with the PIs, the measured TLCs of the two settings were also no different from each other (Table 1). Occurrence of leucocytosis only in tertiary care setting appears to be a reflection of referred severe *P. vivax* malaria cases from other health care settings. Notably, occurrence of leucocytosis has been found to be a risk factor for severe *P. vivax* malaria, prolonged hospitalization, intensive care requirement, and mortality [10,11]. Leucopaenia is more commonly seen in malaria than leucocytosis [12] and thus results in overestimation of PI by assumed TLC of 8,000/μL.

In the recent past, there have been a few studies substantiating the inapplicability of assumed TLC of 8,000/μL in both adult and child populations. In adults, older children and pregnancy, assumed TLC of 8,000/μL has been found to result in significant overestimation of PI, whereas in younger adults it results in marked underestimation [5-7,13]. Thus, in view of the current study and others, it is advisable to abstain using assumed TLC of 8,000/μL for the calculation of PI by microscopy. Actual measurement of patients’ TLC, preferably by automated analyzers or point-of-care portable analyzers, should be made to determine PI for its optimum and reliable application. Alternatively, in resource-poor settings, the malaria population’s mean/median TLCs as applicable should be used for the determination of PI rather than assumed TLC of 8,000/μL. Additionally, one could take on Lambare´ne´ method of PI estimation which does not require either assumed or measured TLC [14] and is as accurate as thin film method.

This is the first study from India, negating the applicability of assumed TLC of 8,000/μL for the calculation of PI in both tertiary care and primary care settings. The study outcomes are based on robust statistical analyses and are in agreement with other studies [5-7]. However, the study lacks a statistically valid sample size, thus, generalizability. Future studies with statistically valid and larger sample size should resolve this issue.

## Conclusions

Assumed TLC of 8,000/μL is not suitable for the calculation of PI. Either actual TLC of the patient should be measured or a representative TLC should be derived for the population under investigation for any study requiring calculated PI by microscopy.
